# On Harmonic Meromorphic Functions Associated with Basic Hypergeometric Functions

**DOI:** 10.1155/2013/164287

**Published:** 2013-10-31

**Authors:** Huda Al dweby, Maslina Darus

**Affiliations:** School of Mathematical Sciences, Faculty of Science and Technology, Universiti Kebangsaan Malaysia, 43600 Bangi, Selangor D. Ehsan, Malaysia

## Abstract

By making use of basic hypergeometric functions, a class of complex harmonic meromorphic functions with positive coefficients is introduced. We obtain some properties such as coefficient inequality, growth theorems, and extreme points.

## 1. Introduction and Preliminaries

Recently, the popularity of the study of basic hypergeometric series (also called *q*-hypergeometric series) is increasing among the researchers. Back in 1748, Euler considered the infinite product (*q*; *q*)_*∞*_^−1^ = ∏_*k*=0_^*∞*^(1 − *q* ^*k*+1^)^−1^ and ever since it became very important to many areas. However, it was stated in the literature that the development of these functions was slower until Heine (1878) converted a simple observation such that lim⁡_*q*→1_[(1 − *q* ^*a*^)/(1 − *q*)] = *a*, which then returns the theory of  _2_ *ϕ*_1_ basic hypergeometric series to the famous theory of Gauss  _2_ *F*_1_ hypergeometric series. Various authors [1–7] introduced classes of analytic functions involving hypergeometric functions and investigated their properties. However, all these results concern mostly the Gaussian and generalized hypergeometric functions. It seems that no attempt has been made to derive similar results for the basic hypergeometric functions. In this work, we proceed to define a class of harmonic meromorphic functions in the unit disk associated with the basic hypergeometric function and discuss some of its properties.

The subject of harmonic univalent functions is a recent area of research which was initially established by Clunie and Sheil-Small [8]. The importance of these functions is due to their use in the study of minimal surfaces as well as in various problems related to applied mathematics and perhaps to other areas of sciences. Hengartner and Schober [9] have introduced and studied special classes of harmonic functions, which are defined on the exterior of the unit disk $\tilde{𝕌}=\{z:|z|>1\}$. They have proved that these functions are complex valued harmonic, sense preserving, univalent mappings *f*, admitting the representation

(1) $$\begin{array}{c} f\left(z\right)=h\left(z\right)+\bar{g\left(z\right)}+A\log\left|z\right|, \end{array}$$

where *h*(*z*) and *g*(*z*) are analytic in $\tilde{𝕌}=\{z:|z|>1\}$.

For *z* ∈ *𝕌*∖{0}, let *M*_*H*_ denote the class of functions

(2) $$\begin{array}{c} f\left(z\right)=h\left(z\right)+\bar{g\left(z\right)}=\frac{1}{z}+\overset{\infty }{\underset{k=1}{\sum }}a_{k}z^{k}+\overset{\infty }{\underset{k=1}{\sum }}b_{k}z^{k}, \end{array}$$

which are harmonic in the punctured unit disk *𝕌*∖{0}, where *h*(*z*) and *g*(*z*) are analytic in *𝕌*∖{0} and *𝕌*, respectively. The class *M*_*H*_ was studied in [10–12].

We further denote by the subclass $M_{\bar{H}}$ of *M*_*H*_ consisting of functions of the form

(3) $$\begin{array}{c} f\left(z\right)=h\left(z\right)+\bar{g\left(z\right)}=\frac{1}{z}+\overset{\infty }{\underset{k=1}{\sum }}a_{k}z^{k}+\bar{\overset{\infty }{\underset{k=1}{\sum }}b_{k}z^{k}},\;\left(a_{k},b_{k}\geq 0\right) \end{array}$$

which are univalent harmonic in the punctured unit disk *𝕌*∖{0}.

A function $f=h+\bar{g}\in M_{H}$ is said to be in the class *MS*_H_* of meromorphically harmonic starlike functions in *𝕌*∖{0} if it satisfies the condition

(4) $$\begin{array}{c} ℜ\left\{-\frac{zh^{\prime }\left(z\right)-\bar{zg^{\prime }(z)}}{h\left(z\right)+g\left(z\right)}\right\}>0,\;\left(z\in 𝕌\setminus \left\{0\right\}\right), \end{array}$$

this class has been studied by Jahangiri and Silverman (see [10]). For harmonic functions

(5) $$\begin{array}{c} f_{j}\left(z\right)=\frac{1}{z}+\overset{\infty }{\underset{k=1}{\sum }}a_{k}^{j}z^{k}+\overset{\infty }{\underset{k=1}{\sum }}\bar{b_{k}^{j}z^{k}},\;j=1,2,\ldots \end{array}$$

the convolution $\left(f_{1}\tilde{*}f_{2})(z\right)$ is defined by

(6) $$\begin{array}{c} \left(f_{1}\tilde{*}f_{2}\right)\left(z\right)=\frac{1}{z}+\overset{\infty }{\underset{k=1}{\sum }}a_{k}^{1}a_{k}^{2}z^{k}+\overset{\infty }{\underset{k=1}{\sum }}\bar{b_{k}^{1}b_{k}^{2}z^{k}}. \end{array}$$

For complex parameters *a*_*i*_, *b*_*j*_, *q*  (*i* = 1,…, *r*,  *j* = 1,…, *s*,  *b*_*j*_ ∈ *ℂ*∖{0, −1, −2,…}), the basic hypergeometric function is defined as follows:

(7) ψ(a1,…,ar;b1,…,bs,q,z)  =∑k=0∞(a1,q)k⋯(ar,q)k(q,q)n(b1,q)k⋯(bs,q)k[(−1)kq(k2)]1+s−rzk,

with $\left(\begin{array}{c} k\\ 2 \end{array}\right)=k(k-1)/2$, where *q* ≠ 0 when *r* > *s* + 1, (*r*, *s* ∈ *ℕ*_0_ = *ℕ* ∪ {0}; *z* ∈ *𝕌*), *ℕ* denotes the set of positive integers, and (*a*, *q*)_*k*_ is the *q*-shifted factorial defined by

(8) $$\begin{array}{c} {\left(a,q\right)}_{k}=\{\begin{array}{cc} 1, & k=0;\\ \left(1-a\right)\left(1-aq\right)\left(1-aq^{2}\right)\cdots \left(1-aq^{k-1}\right), & k\in \mathbb{N}. \end{array} \end{array}$$

By using the ratio test, one recognizes that if 0 < |*q* | <1, the series (7) converges absolutely (see [13]) for all *z* if *r* ≤ *s* and for |*z* | <1 if *r* = *s* + 1.

The *q*-derivative of a function *h*(*x*) is defined by

(9) $$\begin{array}{c} D_{q}\left(h\left(x\right)\right)=\frac{h\left(qx\right)-h\left(x\right)}{\left(q-1\right)x},\;q\neq 1,\;\;x\neq 0. \end{array}$$

For a function *h*(*z*) = *z* ^*k*^, observe that

(10) $$\begin{array}{c} D_{q}\left(h\left(z\right)\right)=D_{q}\left(z^{k}\right)=\frac{1-q^{k}}{1-q}z^{k-1}={\left[k\right]}_{q}z^{k-1}; \end{array}$$

then lim⁡_*q*→1_ *D*_*q*_(*h*(*z*)) = lim⁡_*q*→1_[*k*]_*q*_ *z* ^*k*−1^ = *kz* ^*k*−1^ = *h*′(*z*), where *h*′(*z*) is the ordinary derivative. For more properties of *D*_*q*_, see [14, 15].

Now for *z* ∈ *𝕌*, 0 < |*q* | <1, and *r* = *s* + 1, the basic hypergeometric function defined in (7) takes the form

(11) $$\begin{array}{c} \Psi \left(a_{1},\ldots ,a_{r};b_{1},\ldots ,b_{s},q,z\right)=\overset{\infty }{\underset{k=0}{\sum }}\frac{{\left(a_{1};q\right)}_{k}\cdots {\left(a_{r};q\right)}_{k}}{{\left(q;q\right)}_{k}{\left(b_{1};q\right)}_{k}\cdots {\left(b_{s};q\right)}_{k}}z^{k}‍ \end{array}$$

which converges absolutely in the open unit disk *𝕌*.

Let

(12) Φ([a1,q],z)=zΨ(a1,…,ar;b1,…,bs,q,z)=∑k=0∞∇kr,s([a1,q])zk+1,

where, for convenience,

(13) $$\begin{array}{c} \Phi \left(\left[a_{1},q\right],z\right)=\Phi \left(a_{1},\ldots ,a_{r};b_{1},\ldots ,b_{s},q,z\right),\\ \nabla_{k}^{r,s}\left(\left[a_{1},q\right]\right)=\frac{{\left(a_{1};q\right)}_{k}\cdots {\left(a_{r};q\right)}_{k}}{{\left(q;q\right)}_{k}{\left(b_{1};q\right)}_{k}\cdots {\left(b_{s};q\right)}_{k}}. \end{array}$$

Corresponding to the function Φ([*a*_1_, *q*], *z*), defined in (12), consider

(14) $$\begin{array}{c} \phi_{1}\left(\left[a_{1},q\right],z\right)=\frac{1}{z^{2}}\Phi \left(\left[a_{1},q\right],z\right),\;z\in 𝕌\setminus \left\{0\right\} \end{array}$$

and its inverse function (*ϕ*_1_([*a*_1_, *q*], *z*))^−1^ defined by

(15) $$\begin{array}{c} \phi_{1}\left(\left[a_{1},q\right],z\right)*{\left(\phi_{1}([a_{1},q],z)\right)}^{-1}=\frac{1}{z\left(1-z\right)},\;z\in 𝕌\setminus \left\{0\right\}. \end{array}$$

The series expansion of the inverse function is given as

(16) (ϕ1([a1,q],z))−1=1z+∑k=0∞(q;q)k+1(b1;q)k+1⋯(bs;q)k+1(a1;q)k+1⋯(ar;q)k+1zk=1z+∑k=0∞1∇k+1r,s([a1,q])zk,

where

(17) $$\begin{array}{c} \nabla_{k+1}^{r,s}\left(\left[a_{1},q\right]\right)=\frac{{\left(a_{1};q\right)}_{k+1}\cdots {\left(a_{r};q\right)}_{k+1}}{{\left(q;q\right)}_{k+1}{\left(b_{1};q\right)}_{k+1}\cdots {\left(b_{s};q\right)}_{k+1}}. \end{array}$$

Now, let

(18) H(z)={(ϕ1([a1,q],z))−1−1∇1r,s([a1,q])}=1z+∑k=1∞1∇k+1r,s([a1,q])zk

which is analytic function in the punctured unit disk *𝕌*∖{0}.

Again, corresponding to the function Φ([*a*_1_, *q*], *z*), defined in (12), consider

(19) $$\begin{array}{c} \phi_{2}\left(\left[a_{1},q\right],z\right)=\frac{1}{z}\Phi \left(\left[a_{1},q\right],z\right),\;z\in 𝕌\setminus \left\{0\right\}, \end{array}$$

and its inverse function (*ϕ*_2_([*a*_1_, *q*], *z*))^−1^ defined by

(20) $$\begin{array}{c} \phi_{2}\left(\left[a_{1},q\right],z\right)*{\left(\phi_{2}([a_{1},q],z)\right)}^{-1}=\frac{1}{z\left(1-z\right)},\;z\in 𝕌\setminus \left\{0\right\}. \end{array}$$

The series expansion of the inverse function is given as

(21) (ϕ2([a1,q],z))−1=∑k=0∞(q;q)k(b1;q)k⋯(bs;q)k(a1;q)k⋯(ar;q)kzk=∑k=0∞1∇kr,s([a1,q])zk.

Let

(22) G(z)={(ϕ2([a1,q],z))−1−1∇0r,s([a1,q])}=∑k=1∞1∇kr,s([a1,q])zk,

which is analytic function in *𝕌*. Now, we define the harmonic meromorphic function *F* ∈ *M*_*H*_ as

(23) $$\begin{array}{c} F\left(z\right)=H\left(z\right)+\bar{G(z)}, \end{array}$$

where *H* and *G* are of the forms (18) and (22).

Using convolution $\left(\tilde{*}\right)$ of harmonic meromorphic functions $F(z)=H(z)+\bar{G(z)}$ given by (23) and $f(z)=h(z)+\bar{g(z)}$ given by (2), a linear operator *ℱ*_*s*_^*r*^([*a*_1_, *q*])*f*(*z*) : *M*_*H*_ → *M*_*H*_ is defined as

(24) ℱsr([a1,q])f(z)=F(z)∗~f(z)=H(z)∗h(z)+G(z)∗g(z)¯=1z+∑k=1∞1∇k+1r,s([a1,q])akzk+∑k=1∞1∇kr,s([a1,q])bk¯zk.

Involving the operator *ℱ*_*s*_^*r*^([*a*_1_, *q*]), we introduce the class *M*_*H*_([*a*_1_, *α*, *q*]) as follows.

**Definition 1**

Let *M*_*H*_([*a*_1_, *α*, *q*]) denote the family of harmonic meromorphic functions $f(z)=h(z)+\bar{g(z)}\in M_{H}$ satisfying

(25) |zDq(ℱsr([a1,q])f(z))ℱsr([a1,q])f(z)+1| ≤|zDq(ℱsr([a1,q])f(z))ℱsr([a1,q])f(z)+2α−1|.

Equivalently,

(26) |zDq(H(z)∗h(z))+zDq(G(z)∗g(z))¯H(z)∗h(z)+G(z)∗g(z)¯+1|  ≤|zDq(H(z)∗h(z))+zDq(G(z)∗g(z))¯H(z)∗h(z)+G(z)∗g(z)¯+2α−1|,

for some *α*  (0 ≤ *α* < 1) and for all *z* ∈ *𝕌*∖{0}.

For *r* = 1, *s* = 0, *a*_1_ = *q*, and *q* → 1, *M*_*H*_([*q*, *α*, *q* → 1]) is the class of harmonic meromorphic starlike functions of order *α* and for *r* = 1, *s* = 0, *a*_1_ = *q*, *α* = 0, and *q* → 1, the class *M*_*H*_([*q*, 0, *q* → 1]) gives the class of harmonic meromorphic starlike functions for all *z* ∈ *𝕌*∖{0}.

Denote $M_{\bar{H}}([a_{1},\alpha ,q])=M_{H}([a_{1},\alpha ,q])\cap M_{\bar{H}}$.

## 2. Main Results

**Theorem 2**

If $f=h+\bar{g}$ is of the form (2) and satisfies the condition

(27) ∑k=1∞q([k]q+α){1∇k+1r,s([a1,q])|ak|+1∇kr,s([a1,q])|bk|}  ≤1−qα,

where (0 ≤ *α* < 1, |*q* | <1), then *f* is harmonic univalent sense preserving in *𝕌*∖{0} and *f* ∈ *M*_*H*_([*a*_1_, *α*, *q*]).

**Proof**

Suppose that (27) holds true for 0 ≤ *α* < 1. Consider the expression

(28) A(z)=|zDq(H(z)∗h(z))+zDq(G(z)∗g(z))¯  +(H(z)∗h(z))+(G(z)∗g(z))¯|−|zDq(H(z)∗h(z))+zDq(G(z)∗g(z))¯   +(2α−1)(H(z)∗h(z))   +(2α−1)(G(z)∗g(z))¯|,A(z)=|q−1qz+∑k=1∞([k]q+1) ×[1∇k+1r,s([a1,q])akzk+1∇kr,s([a1,q])bkzk¯]|−|q(2α−1)−1qz+∑k=1∞([k]q+2α−1)   ×[1∇k+1r,s([a1,q])akzk+1∇kr,s([a1,q])bkzk¯]|,A(r)≤q−1qr+∑k=1∞([k]q+1)×[1∇k+1r,s([a1,q])|ak|+1∇kr,s([a1,q])|bk|]rk−(−q(2α−1)+1qr)+∑k=1∞([k]q+2α−1)×[1∇k+1r,s([a1,q])|ak|+1∇kr,s([a1,q])|bk|]rk=∑k=1∞2([k]q+α) ×[1∇k+1r,s([a1,q])|ak|    +1∇kr,s([a1,q])|bk|]rk−2(1−qα)qr.

That is,

(29) qrA(r)≤∑k=1∞2q([k]q+α) ×[1∇k+1r,s([a1,q])|ak|   +1∇kr,s([a1,q])|bk|]rk+1−2(1−qα).

The inequality in (28) holds true for all *r*  (0 ≤ *r* < 1). Therefore, letting *r* → 1 in (28), we obtain

(30) qA(r)≤∑k=1∞2q([k]q+α) ×[1∇k+1r,s([a1,q])|ak|+1∇kr,s([a1,q])|bk|] −2(1−qα).

By hypothesis (27), it follows that (26) holds, so that *f* ∈ *M*_*H*_([*a*_1_, *α*, *q*]). Note that *f* is sense preserving in *𝕌*∖{0}. This is because

(31) |Dqh(z)|≥1r2−∑k=1∞[k]q|ak||r|k−1>1−∑k=1∞[k]q|ak|≥1−∑k=1∞q([k]q+α)1−α1∇k+1r,s([a1,q])|ak|≥∑k=1∞q([k]q+α)1−α1∇kr,s([a1,q])|bk|≥∑k=1∞[k]q|bk|>∑k=1∞[k]q|bk|rk−1≥|Dqg(z)|.

Then, we have lim⁡_*q*→1_[|*D*_*q*_ *h*(*z*)|≥|*D*_*q*_ *g*(*z*)|] = [|*h*′(*z*)|≥|*g*′(*z*)|]. Hence the theorem.

Now, we prove that condition (27) is necessary for functions in $M_{\bar{H}}([a_{1},\alpha ,q])$.

**Theorem 3**

Let $f=h+\bar{g}\in M_{\bar{H}}$ be a function defined by (3). Then, $f\in M_{\bar{H}}([a_{1},\alpha ,q])$ if and only if the inequality

(32) ∑k=1∞q([k]q+α){1∇k+1r,s([a1,q])ak+1∇kr,s([a1,q])bk}  ≤1−qα

is satisfied.

**Proof**

In view of Theorem 2, it suffices to show that the “only if" part is true. Assume that $f\in M_{\bar{H}}([a_{1},\alpha ,q])$. Then,

(33) |(zDq(H(z)∗h(z))+zDq(G(z)∗g(z))¯H(z)∗h(z)+G(z)∗g(z)¯+1) ×(zDq(H(z)∗h(z))+zDq(G(z)∗g(z))¯H(z)∗h(z)+G(z)∗g(z)¯+2α−1)−1|=|(q−1qz+∑k=1∞([k]q+1)[1∇k+1r,s([a1,q])akzk+1∇kr,s([a1,q])bkzk¯])  ×(q(2α−1)−1qz  +∑k=1∞([k]q+2α−1)[1∇k+1r,s([a1,q])akzk  +1∇kr,s([a1,q])bkzk¯])−1|≤1, z∈𝕌∖{0}.

Since *ℜ*(*z*)≤|*z*| for all *z*, it follows from (33) that

(34) ℜ{(q−1qz+∑k=1∞([k]q+1)[1∇k+1r,s([a1,q])akzk+1∇kr,s([a1,q])bkzk¯])  ×(q(2α−1)−1qz    +∑k=1∞([k]q+2α−1)[1∇k+1r,s([a1,q])akzk    +1∇kr,s([a1,q])bkzk¯])−1}  ≤1, z∈𝕌∖{0}.

We now choose the values of *z* on the real axis. Upon the clearing the denominator in (34) and letting *z* → 1 through real values, we obtain the following:

(35) ∑k=1∞([k]q+1)[1∇k+1r,s([a1,q])akzk       +1∇kr,s([a1,q])bkzk¯] ≤2(1−qα)q−∑k=1∞([k]q+2α−1)  ×[1∇k+1r,s([a1,q])akzk    +1∇kr,s([a1,q])bkzk¯],

which immediately yields the required condition (32).

Next, we consider a distortion property for functions in the class $M_{\bar{H}}([a_{1},\alpha ,q])$ as follows.

**Theorem 4**

If $f=h+\bar{g}$ defined by (3) is in the class $M_{\bar{H}}([a_{1},\alpha ,q])$, then for *a*_1_ = *q*,  ∑(*b*_*j*_ − *a*_*i*_) > 1, where *i* = {2,…, *r*}, *j* = {1,…, *s*}, and 0 < |*z* | = *r* < 1, one has

(36) 1r−1q(1−qα1+α)(∏i=2r(1−ai)∏j=1s(1−bj))r  ≤|f(z)|≤1r+1q(1−qα1+α)(∏i=2r(1−ai)∏j=1s(1−bj))r.

**Proof**

We will only prove the right-hand inequality. The proof for the left-hand inequality is similar and we will omit it:

(37) |f(z)|≤|1z+∑k=1∞akzk+∑k=1∞bkzk¯|≤1r+∑k=1∞|ak|rk+∑k=1∞|bk|rk¯≤1r+r∑k=1∞(|ak|+|bk|)≤1r+1q(1−qα1+α)∇1r,s([a1,q])×∑k=1∞(q([k]q+α)1−qα)   ×1∇kr,s([a1,q])(|ak|+|bk|)r≤1r+1q(1−qα1+α)∇1r,s([a1,q])×∑k=1∞(q([k]q+α)1−qα)  ×[1∇k+1r,s([a1,q])|ak|    +1∇kr,s([a1,q])|bk|]r≤1r+1q(1−qα1+α)(∏i=2r(1−ai)∏j=1s(1−bj))r.

The functions

(38) $$\begin{array}{c} f\left(z\right)=\frac{1}{z}+\frac{1}{q}\left(\frac{1-q\alpha }{1+\alpha }\right)\left(\frac{\prod_{i=2}^{r}\left(1-a_{i}\right)}{\prod_{j=1}^{s}\left(1-b_{j}\right)}\right)z,\\ f\left(z\right)=\frac{1}{z}+\frac{1}{q}\left(\frac{1-q\alpha }{1+\alpha }\right)\left(\frac{\prod_{i=2}^{r}\left(1-a_{i}\right)}{\prod_{j=1}^{s}\left(1-b_{j}\right)}\right)\bar{z} \end{array}$$

for 0 ≤ *α* < 1 show that the bounds given in Theorem 4 are sharp in *𝕌*∖{0}.

Next, we give the following.

**Theorem 5**

Set

(39) h0(z)=1z,hk(z)=1z+1q(1−qα[k]q+α)∇k+1r,s([a1,q])zk,k=1,2,…,g0(z)=0,gk(z)=1q(1−qα[k]q+α)∇kr,s([a1,q])zk¯,k=1,2,….

Then, $f\in M_{\bar{H}}\left(\left[a_{1},\alpha ,q\right]\right)$, if and only if, it can be expressed in the form

(40) $$\begin{array}{c} f\left(z\right)=\overset{\infty }{\underset{k=0}{\sum }}\left(\lambda_{k}h_{k}+\gamma_{k}g_{k}\right), \end{array}$$

where *λ*_*k*_ ≥ 0, *γ*_*k*_ ≥ 0, and ∑_*k*=0_^*∞*^(*λ*_*k*_ + *γ*_*k*_) = 1. In particular, the extreme points of $M_{\bar{H}}([a_{1},\alpha ,q])$ are {*h*_*k*_} and {*g*_*k*_}.

**Proof**

Let

(41) f(z)=∑k=0∞(λkhk+γkgk)=∑k=0∞(λk+γk)1z+(1−qαq([k]q+α))×∑k=0∞∇k+1r,s([a1,q])λkzk+∑k=0∞∇kr,s([a1,q])γkzk¯.

Then,

(42) ∑k=0∞(q([k]q+α)1−qα)1∇k+1r,s([a1,q])   ×(1−qαq([k]q+α)∇k+1r,s([a1,q])λk)   +∑k=0∞(q([k]q+α)1−qα)1∇kr,s([a1,q])   ×(1−qαq([k]q+α)∇kr,s([a1,q])γk)  =∑k=1∞(λk+γk)=1−λ0−γ0≤1.

So, $f\in M_{\bar{H}}([a_{1},\alpha ,q])$. Conversely, suppose that $f\in M_{\bar{H}}([a_{1},\alpha ,q])$. Set

(43) $$\begin{array}{c} \lambda_{k}=\left(\frac{q\left({\left[k\right]}_{q}+\alpha \right)}{1-q\alpha }\right)\frac{1}{\nabla_{k+1}^{r,s}\left(\left[a_{1},q\right]\right)}a_{k},\;k\geq 1,\\ \gamma_{k}=\left(\frac{q\left({\left[k\right]}_{q}+\alpha \right)}{1-q\alpha }\right)\frac{1}{\nabla_{k}^{r,s}\left(\left[a_{1},q\right]\right)}b_{k},\;k\geq 0. \end{array}$$

Then, by Theorem 3, 0 ≤ *λ*_*k*_ ≤ 1 (*k* = 1,2, 3,…) and 0 ≤ *γ*_*k*_ ≤ 1 (*k* = 0,1, 2,…).

(44) $$\begin{array}{c} \text{We define }\lambda_{0}=1-\overset{\infty }{\underset{k=1}{\sum }}\lambda_{k}-\overset{\infty }{\underset{k=0}{\sum }}\gamma_{k}, \end{array}$$

and note that, by Theorem 3, *λ*_0_ ≥ 0. Consequently, we obtain

(45) $$\begin{array}{c} f\left(z\right)=\overset{\infty }{\underset{k=0}{\sum }}\left(\lambda_{k}h_{k}+\gamma_{k}g_{k}\right). \end{array}$$

This proves the theorem.

**Remark 6**

Other work related to *q*-hypergeometric functions and analytic functions can be found in [16, 17].
